# Operator bias in software-aided bat call identification

**DOI:** 10.1002/ece3.1122

**Published:** 2014-05-30

**Authors:** Georg Fritsch, Alexander Bruckner

**Affiliations:** Institute of Zoology, University of Natural Resources and Life SciencesVienna, Austria

**Keywords:** Acoustic identification, automated classification, batcorder, identification bias, observer experience

## Abstract

Software-aided identification facilitates the handling of large sets of bat call recordings, which is particularly useful in extensive acoustic surveys with several collaborators. Species lists are generated by “objective” automated classification. Subsequent validation consists of removing any species not believed to be present. So far, very little is known about the identification bias introduced by individual validation of operators with varying degrees of experience. Effects on the quality of the resulting data may be considerable, especially for bat species that are difficult to identify acoustically. Using the batcorder system as an example, we compared validation results from 21 volunteer operators with 1–26 years of experience of working on bats. All of them validated identical recordings of bats from eastern Austria. The final outcomes were individual validated lists of plausible species. A questionnaire was used to enquire about individual experience and validation procedures. In the course of species validation, the operators reduced the software's estimate of species richness. The most experienced operators accepted the smallest percentage of species from the software's output and validated conservatively with low interoperator variability. Operators with intermediate experience accepted the largest percentage, with larger variability. Sixty-six percent of the operators, mainly with intermediate and low levels of experience, reintroduced species to their validated lists which had been identified by the automated classification, but were finally excluded from the unvalidated lists. These were, in many cases, rare and infrequently recorded species. The average dissimilarity of the validated species lists dropped with increasing numbers of recordings, tending toward a level of ˜20%. Our results suggest that the operators succeeded in removing false positives and that they detected species that had been wrongly excluded during automated classification. Thus, manual validation of the software's unvalidated output is indispensable for reasonable results. However, although application seems easy, software-aided bat call identification requires an advanced level of operator experience. Identification bias during validation is a major issue, particularly in studies with more than one participant. Measures should be taken to standardize the validation process and harmonize the results of different operators.

## Introduction

Identification of species is a crucial step in many ecological studies; its accuracy may heavily influence the quality of the data. Accuracy and consistency are especially critical if results from several people are compiled into the same data set, for example, in extensive surveys and monitoring. For various plant and animal groups, identification results can vary considerably, even among acknowledged experts, due to so-called observer or operator bias (e.g., McClintock et al. 2010; Farmer et al. 2012; Miller et al. 2012). In research on bias in identification from acoustic records (mostly of anurans, birds, and insects), false positives (species not present, but wrongly censused or identified) are distinguished from false negatives (species present, but not censused or misidentified). In summarizing other studies, Farmer et al. (2012) and Miller et al. (2012) conclude that false positives have so far largely been ignored in ecological studies, but may significantly influence results and conclusions. For anuran call surveys, Lotz and Allen (2007) found that false positives contribute much more to the observer bias than false negatives.

The frequency, duration, and modulation of bat calls can be used to distinguish among species. In recent years, a number of largely automated tools for recording and identifying bat calls have been developed that combine hardware and software components, for example, the batcorder (Ecoobs, Nuremberg, Germany, http://www.ecoobs.de), Batlogger (Elekon AG, Luzern, Switzerland, http://www.batlogger.ch; Obrist et al. 2004), and iBatID (https://sites.google.com/site/ibatsresources/iBatsID; Walters et al. 2012). They speed up and facilitate the complex task of acoustic identification and have become increasingly popular (Runkel 2008; Müller et al. 2012; Plank et al. 2012; Frey-Ehrenbold et al. 2013). Automated tools are expected to reduce workloads considerably and, because their software incorporates expert knowledge, to deliver a standardized or “objective” approach to identification (Obrist et al. 2004; Acevedo et al. 2009; Marckmann and Runkel 2010b). However, as a proportion of the calls may be misidentified, the output of the automated classification requires validation. Validation consists of removing from species lists any species not believed to be present and, in some cases, of adding species to the list. Operators may not be consistent in their validation, so that bias may be introduced into “objectively” classified data. This may be especially severe for species that are difficult or almost impossible to discriminate acoustically (e.g., several *Myotis* species, Russo and Jones 2002; Obrist et al. 2004).

We quantified operator bias within a volunteer group of 21 bat specialists who were individually assigned 12 identical sets of bat recordings for validation. The global research question we asked was as follows: How large is the variability between the validated results of the specialists? Specifically, we asked: Are validated results influenced by operator experience? Does the number of recordings influence the variability of validated results? What is the significance for the identification of individual species? What is the practical implication of the (dis)similarity of the validated species assemblages?

## Materials and Methods

### Field recordings

From a batcorder survey of 105 sites in eastern Austria in summer 2010 (Fritsch G. & Kubista C., unpubl. data), we selected 12 sites randomly, so that the entire data set (3459 call sequences) could be processed by an operator with approximately 1 day's work. At each site, recordings were made automatically by one batcorder over one night (7 pm–6 am). We used batcorder models V1 (three sites) and V2 (nine sites), with the manufacturer's default settings (quality 20, threshold −27db, posttrigger 600 ms, critical frequency 16 kHz). In accordance with the manufacturer's recommendations (Ecoobs 2010), the batcorders were mounted on tent poles approximately 2 m above ground and at a minimum distance of 2 m from vegetation.

The recordings covered a range of bat activity and species. The number of call sequences recorded per site ranged from 11 to 1429 (median: 73.5). The number of species identified by batIdent ranged from 2 to 14 (median: 7) per site.

### Batcorder and software-aided identification

The “batcorder system” consists of three components that operate as a working unit: the recording device “batcorder,” the administration/visualization software “bcAdmin,” and the identification software “batIdent”. bcAdmin (Marckmann and Runkel 2010a) discerns individual bat calls within the recorded sound and measures their various acoustical properties (call length, maximum and minimum frequency, etc.; Runkel 2010). The measurements are fed into batIdent where the individual calls are identified using Random Forest classifications by R statistical software (Marckmann and Runkel 2010b). Ideally, this procedure continues to the terminal category of the decision tree, that is, to species level. Finally, based on the classifications of its constituent calls, each call sequence is assigned an overall classification. For long sequences containing many calls, a second and even a third classification may be assigned. Thus, the output of the automated classification is an unvalidated list of call sequences, each assigned one to three taxa.

Currently, batIdent is able to discriminate 22 European species (Runkel 2010). Two additional terminal classifications, “Plecotus” and “Mbart,” each comprise more than one species (*Plecotus* spp., and *Myotis mystacinus/brandtii,* respectively) that cannot be discriminated reliably. They are treated as operational taxonomic units (OTU) at the same level as species. As a third OTU, we introduced “Pmid” (*Pipistrellus* middle frequency; *P. nathusii/kuhlii*). Although batIdent can discriminate these two species, we followed here the approach of the majority of the operators (17 of 21).

Below, we use the term “species” to refer to all terminal categories of batIdent's decision tree: proper species as well as the OTUs Plecotus, Mbart, and Pmid. Intermediate classifications (nodes of the decision tree) have not been included in the analysis of the validation results. They have no practical significance and, according to our experience, are not commonly listed in validated outputs.

### Operators and questionnaire

In 2011, we contacted all persons and organizations in Germany, Austria, and Switzerland we knew to use the batcorder system, asking them to participate in our study and to suggest further candidates. This way we compiled a list of 63 potential volunteer operators. We are confident that we reached most of the “batcorder community” in German-speaking Central Europe.

Every operator validated the same batcorder recordings (.raw files) from the 12 sites. Before validation, the operators executed initial classifications using bcAdmin and batIdent (v1.0 or higher), generating unvalidated species lists for each site. These were identical for all 21 operators. After validation, the output was a list of plausible validated species for each site, plus a completed questionnaire comprising information about the operator and his/her validation procedure (details below).

In addition to the recordings, operators were provided with supplements for site characterization to aid in assessing the occurrence probability of species: an aerial photograph, a detail from a geographic map (scale 1:50,000), site photographs, field notes on site characteristics (e.g., coverage and spatial structure of vegetation, proximity to human settlements), and records of ambient temperature taken every 15 min throughout the night in which the calls were recorded.

The questionnaire consisted of two parts (see Appendix S1). In Part 1, operators were asked about their experience: (1) number of years spent working on bats; (2) experience of acoustic analysis of bat calls in general (measured as the number of nights analyzed); (3) experience of the batcorder system previous to this study (number of nights validated). In Part 2, operators detailed their validation procedure: (1) how important they felt the supplements for site characterization were; (2) how frequently they used the various functions of batIdent and bcAdmin (e.g., graphical representation of single calls, decision tree diagram); (3) which other resources were consulted (e.g., other sound analysis software, personal records, and literature); and (4) the software versions of bcAdmin, batIdent, and R they used (including potential modifications of the default settings). Answers to questions (1) to (3) in Part 2 were given on an ordinal scale from 1 (not important) to 5 (very important).

All responses were anonymized by a person not involved in the study; operators' names were replaced with identifiers O1 to O21.

### Analysis

Univariate analyses to describe the operators, validation results, and correlations were performed basically with graphical methods, using R v2.15 (R Core Team 2012).

To differentiate experience levels, we subjected the three experience-related operator traits to a correlation-based principal components analysis (PCA).

Multivariate analysis of the relative variability of the resulting species lists was based on Sørensen's similarity of the individual operator's results at each recording site. On that we ran nonmetric multidimensional scaling (NMDS) to see from the scatter at each recording site if species lists could be differentiated.

The variability can also be formally described as heterogeneity in the multivariate dispersion of the 21 validated species lists of each site. We used PERMANOVA's PERMDISP routine to measure this heterogeneity. PERMDISP calculates in multivariate space the distances *z*_*ij*_ of the species list of each operator *i* and site *j* to the respective site centroid (i.e., the centroid of the 21 species lists the operators produced for that site; see Anderson 2006; Anderson et al. 2008, 87ff). Hence, sites for which very different validated lists were submitted by the operators scored high distance values.

Subsequently, we used this distance as a univariate measure for dissimilarity and checked for correlation with the number of call sequences. The multivariate analysis was carried out in PRIMER v6.1.12 (Clarke and Gorley 2006) and the add-on PERMANOVA v1.0.2 (Anderson et al. 2008).

## Results

Results from 21 people were returned by the end of 2011. All had previous experience with acoustic analysis of bats as graduate students, researchers, or environmental consultants.

### Operator's experience in bat work

**Figure 1:**
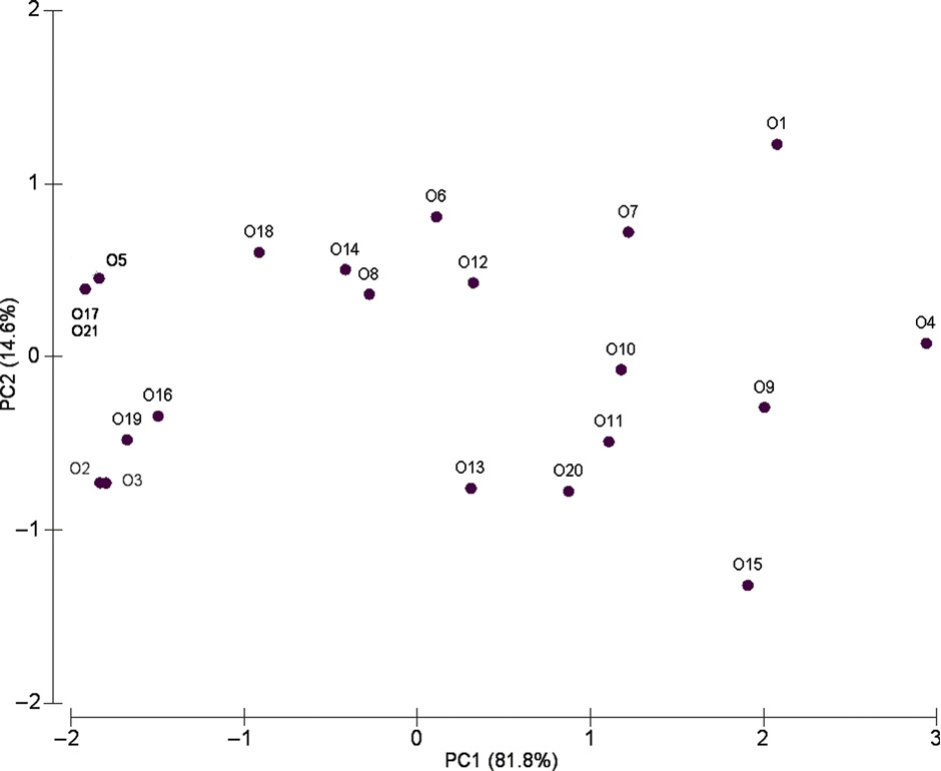
Principal component analysis of three working experience traits in a study on operator bias in bat call identification. Experience traits: (i) number of years working on bats; (ii) experience in analyzing bat calls, regardless of method used; and (iii) experience with the batcorder system before this study. Operators are labeled O1–O21. The gradient of experience ranges along PC1 from least experienced (left) to most experienced (right).

Operators had worked on bats for 1 to 26 years (median: 3). Their level of experience in analyzing bat calls with generic acoustical methods ranged from 15 to 800 nights (median: 215). Previous experience with the batcorder system ranged from 0 to 600 validated nights (median: 96). The PCA performed with these three traits produced a ranking of experience. As the first PC comprised 82% of total variability, we used the operators' scores on PC1 as a summarizing variable for “operator experience” in subsequent analyses. PC1 represented a gradient from least experienced to most experienced operators (Fig. 1, left to right). Although the PCA revealed no distinct separation between groups, we adopted tripartite, descriptive categories, as widely used in the literature (e.g., Farmer et al. 2012). Operators classed as “intermediate” in experience had 2–18 years of experience of working on bats and had analyzed calls with generic acoustical methods recorded on 200–400 nights. Least experienced and most experienced operators had less and more working experience, respectively.

### Operator's individual validation procedure

The time operators spent for validation of all 12 sites together ranged from 108–650 min (median 376). Validation times for single sites ranged from 1–20 min (median 5, site 6) to 12–190 min (median 45, site 12). There was no correlation between validation time and experience (PC1 scores). Validation time and resulting number of species were only weakly correlated (Pearson's r = 0.50; P < 2.2e–16).

**Figure 2:**
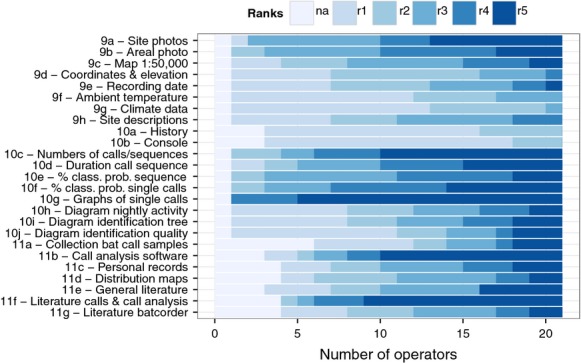
Questionnaire ratings of supporting resources used in the individual validation procedure of 21 operators in a study on operator bias in bat call analysis. Ratings were from r1 (light blue, not important) to r5 (dark blue, very important), “na”: no answer given. Three sets of questions dealt with: supplements provided for site characterization (9a–h), functions of the identification software bcAdmin and batIdent (10a–j), and other resources used by the operators (11a–g).

No supplement provided for site characterization, software function, or other resource was considered unimportant (rank 1) for species validation by all of the operators (Fig. 2).

Of the supplements provided for site characterization, site photographs (9a) were considered the most important, followed by aerial photographs (9b) and geographic maps (9c). Of least importance were geographic coordinates and elevation, and the climate and night temperature data.

Of the software functions, three achieved a rating distinctly above average: graphical display of single calls (10g), number of calls per call sequence (10c), and classification probability of calls (10f). Features of bcAdmin which provided overviews of identifications and activity for each site (10h, i, j) or gave low-level access to the batIdent classification procedure (10a, b) were rarely used (Fig. 2).

Of the other resources used by the operators, sound analysis software other than bcAdmin/batIdent (11b) and bat call literature (11f) stood out as most important. The intermediate and most experienced operators also used their own records (11c). Collections of call samples (11a) and distribution maps (11d) scored high, mainly among the least experienced operators (Fig. 2).

The versions of batIdent used ranged from v1.01 to v1.03, so that the batIdent outputs used as the basis for subsequent validation were identical for all operators.

### Validation results and number of recordings

**Figure 3:**
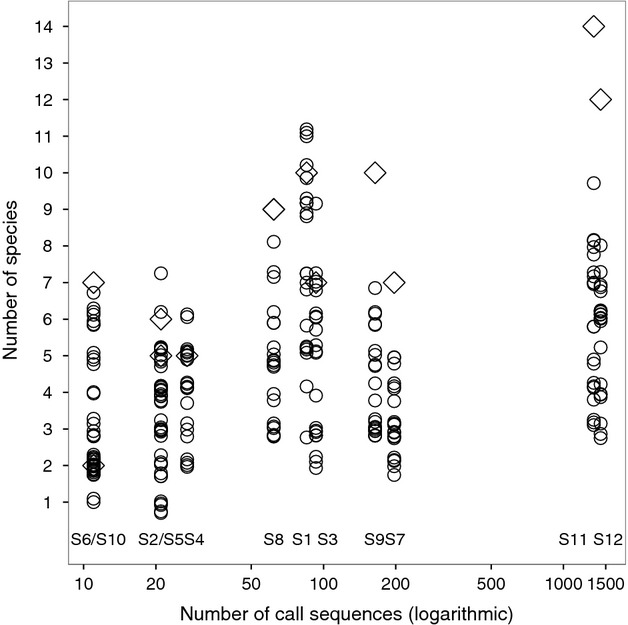
Relationship between the number of recorded call sequences per study site and (1) the number of unvalidated species from bat call identification software (diamonds), (2) the number of validated species produced by 21 operators (circles), in a study on operator bias in bat call identification. Each vertical strip represents a site (S1 to S12). Note the logarithmic scale of the abscissa. On two occasions, the same number of call sequences were recorded in two sites (S6/S10, S2/S5; 11 and 21 sequences).

The number of species listed in the unvalidated output from batIdent increased monotonically and significantly with the number of call sequences (Pearson's r = 0.76, P = 0.004; diamonds in Fig. 3). But we did not find a similar correlation between the number of call sequences and the median number of validated species. While the accumulation curve of the number of unvalidated species (diamonds) did not seem to reach an asymptote even after 1500 call sequences, the number of validated species (circles) increased less steadily above ∼100 call sequences.

Generally, for each site, the number of unvalidated species in the batIdent output was higher than the number of validated species (Fig. 3). The difference between unvalidated and validated species lists was smaller in sites with low bat activity (few call sequences) than in sites with high bat activity. Only some of the least and intermediate experienced operators produced a validated species list that was longer than the unvalidated list (sites 1–4).

### Validation results and experience

**Figure 4:**
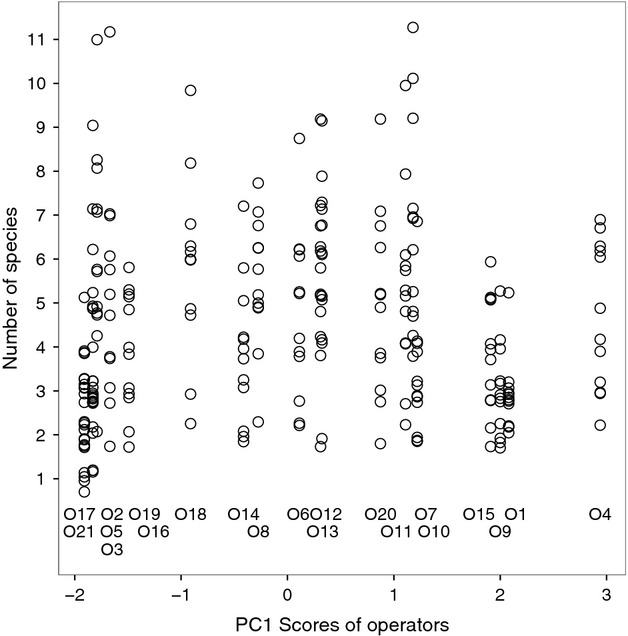
Variability in validation of species lists by 21 operators in a study on operator bias in bat call identification. Each vertical strip represents an operator (O1 to O21), each circle one of 12 sites. Operators were ranked along the abscissa from left (least experienced) to right (most experienced), using principal component analysis scores for a synthetic experience variable (Fig. 1).

The most experienced operators accepted on average the smallest percentage of species from the unvalidated list produced by batIdent (median: 43%). Their validation behavior was consistent: It exhibited the smallest variation among operators (Fig. 4). They rarely identified additional species.

The large group of operators with intermediate experience accepted on average the highest proportion (median: 71%) of species from the unvalidated list and were also most likely to identify additional species (Table 1). There was higher within-group variability in their validation behavior than in that of the most experienced operators (Fig. 4).

**Table 1:** Identification of species in a study on operator bias in bat call analysis. Numbers of sites (of a maximum of 12) in which each species was identified by automated identification software (batIdent: species unvalidated), and numbers of sites in which identification of each species was accepted by 21 operators (O1–O21: species validated). Parenthesized bold font: Number of times an operator listed a species which was not included in batIdent's overall classifications for whole call sequences, but which had been identified from single calls (“additional species”). Operators are ranked by experience (scores on PC1, Fig. 1) from least (left) to most experienced (right)

	batIdent	O21	O17	O5	O2	O3	O19	O16	O18	O14	O8	O6	O13	O12	O20	O11	O10	O7	O15	O9	O1	O4
Ppip	10	9	8	9	9	10	10	9	10	9	10	10	10	10	11 (**1**)	10	10	10	10	10	10	9
Bbar	5	3	4	2	4	4	4	3	4	5	4	4	4	5	4	4	4	4	3	4	4	3
Nnoc	9	6	7	4	6	8	8	6	8	3	8	8	9	9	7	7	9 (**1**)	5	7	8	7	7
Ppyg	3	2	2	2	3	3	3	2	3	3	3	3	3	3	4 (**1**)	3	2	2	3	3	1	2
Mdau	9	1	1	6	6	9 (**1**)	6	7	9	8	8	6	9	9	6	8	8	5	4	2	4	9
Mbart	10	3	3	1	9	10	5	8	10	3	6	9	10	9	7	8	11 (**1**)	6	8		2	8
Pmid	6	3 (**1**)	2	5 (**2**)	1		7 (**2**)	4 (**1**)	8 (**2**)	6 (**2**)	7 (**2**)	5 (**1**)	6 (**1**)		8 (**2**)	6 (**2**)		5 (**2**)		7 (**3**)	4 (**2**)	7 (**2**)
Mnat	3				2	3	2		2		4 (**1**)	1	3	3	1	1	3 (**1**)	1	2			2
Hsav	3					2 (**1**)	1		1	1	1		1	1	1		2 (**1**)			1		1
Eser	3				3 (**1**)	5 (**3**)	5 (**3**)		2 (**1**)	4 (**1**)	3 (**1**)	2	2	3	3	6 (**3**)	2		2			2
Mbec	7	1	1		3	4	3	2	5	1	1	2		4	1	3	3				1	
Enil	2	1			2 (**1**)	1	4 (**2**)	1	1	1	1	1	1	1	1	1	2 (**1**)	1			1	1
Mema	4	1			3	6 (**2**)	4 (**2**)	2	3	2	4	2	3	3	1	2	7 (**4**)				1	2
Mmyo	2	1				1	1	1	1		3 (**2**)	1	2 (**1**)	1	2 (**1**)	3 (**2**)	3 (**1**)		2 (**1**)			1
Malc	3				1				2		1			1	2	1						
Plec	0										(**1**)				(**1**)	(**1**)	(**2**)	(**1**)				(**1**)
Pkuh	5				4	5		1						2			5					
Pnat	3				1	3 (**1**)						2 (**1**)		3			5 (**2**)		5 (**2**)	1		
Nlei	2										1		1	2			(**2**)					(**1**)
Vmur	0								(**1**)				(**2**)									
Mdas	1													1								

Ppip, *Pipistrellus pipistrellus*; Bbar, *Barbastella barbastellus*; Nnoc, *Nyctalus noctula*; Ppyg, *P. pygmaeus*; Mdau, *M. daubentonii*; Mbart, *M. mystacinus/brandtii*; Pmid, *P. kuhlii/nathusii*; Mnat, *M. nattereri*; Hsav, *Hypsugo savii*; Eser, *E. serotinus*; Mbec, *M. bechsteinii*; Enil, *Eptesicus nilssonii*; Mema, *M. emarginatus*; Mmyo, *Myotis myotis*; Malc, *M. alcathoe*; Plec, *Plecotus* spp.*; Pkuh*, *P. kuhlii; Pnat*, *P. nathusii;* Nlei, *N. leisleri*; Vmur, *Vespertilio murinus*; and Mdas, *M. dasycneme*.

Among the least experienced operators, we observed the most variable validation behavior: Some were extremely cautious in their validation (listing only 1–3 species per site in their validated lists); some were very confident (listing up to 11 species per site; Fig. 4).

### Validation of additional species

For long sequences containing many calls, for which a second and sometimes a third species classification had been assigned by batIdent, the operators did not include these species in their validated lists. Rather, they always ignored these second and third classifications.

However, 14 operators (66%) added species to their validated lists that were not included in the unvalidated species list produced by batIdent. These operators scrutinized the automated identifications of individual calls and validated species assigned to calls interspersed within the sequences. They extended the species list of at least one site by adding these, subsequently called, “additional species” (Table 1, bold font).

### Validation of individual species

*Pipistrellus pipistrellus* and *Barbastella barbastellus* were accepted in 100% and 80% of all cases, respectively (medians, % of acceptance of the batIdent classifications, Table 1), independent of operator experience. This similarly applied to *Nyctalus noctula*, *P. pygmaeus,* and *M. daubentonii* (medians 78%, 100%, 67%), although for these species there was more variability among the operators. The operators validated all of them with high confidence, even when only a few call sequences of the species were recorded. This corresponds well with Hammer et al. (2009) and partly with Jennings et al. (2008), who rated the identification of these species as straightforward.

Acceptance of Mbart (*M. mystacinus/brandtii*) was equally high (median 80% of all batIdent identifications), but with a much larger variability among the operators.

*Myotis myotis*, *M. emarginatus*, *M. bechsteinii*, *M. nattereri*, *Eptesicus nilssonii*, *E. serotinus,* and *Hypsugo savii* were less frequently and less unanimously accepted during validation (medians ranged 14% Mbec –68% Eser). For sites with numerous recordings, they were accepted by the majority of operators, for sites with lower bat activity only by a few. In the latter case, they mostly emerged as additional species (predominantly *M. emarginatus, M. myotis,* and *E. nilssonii*). The most and the least experienced operators were less likely to accept these species during validation than operators with intermediate experience (Table 1).

*M. alcathoe*, *M. dasycneme, V. murinus*, *P. nathusii*, *P. kuhlii*, *Plecotus* spp., and *N. leisleri* were accepted only a few times by single operators.

For *P. nathusii*/*kuhlii,* we found a diverging pattern: The majority of operators (17 of 21) did not trust the discrimination of these acoustically similar species by batIdent and pooled them into the generic OTU Pmid in their validated lists. In contrast, some operators listed *P. nathusii* several times as an additional species (Table 1).

*Vespertilio murinus* and *Plecotus* spp. were listed several times, although they were not in the unvalidated batIdent output of any site.

Occasionally, species listed in the batIdent output were unanimously rejected by all operators. All three classifications of *Miniopterus schreibersi* and one of *Tadarida teniotis* probably because they were considered to be misclassifications by the software and outside their distribution area.

*H. savii*, *M. alcathoe,* and *M. bechsteinii* were also rejected by all operators at sites where they were represented by only a few, doubtful call sequences.

### (Dis)similarity of validated species lists

**Figure 5:**
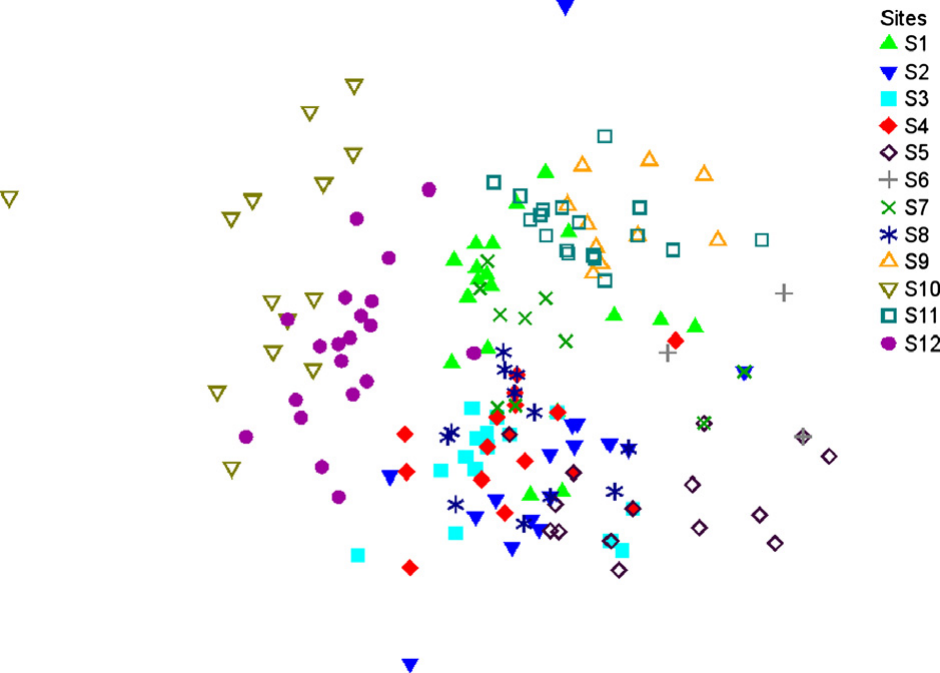
Nonmetric multidimensional scaling (NMDS, stress 0.19) of species lists for 12 study sites produced by 21 operators in a study on operator bias in bat call analysis. Each dot represents a validated species list produced by an operator. Not all dots are visible, due to manifold overplotting.

Nonmetric multidimensional scaling (NMDS) of the 252 validated species lists (produced by 21 operators for each of the 12 sites) revealed considerable variability among the species lists of a site (the scatter in Fig. 5). That is, the species lists produced by the operators differed widely. Nevertheless, and despite much overlap of the dots in Fig. 5, it was possible to distinguish almost all sites clearly.

**Figure 6:**
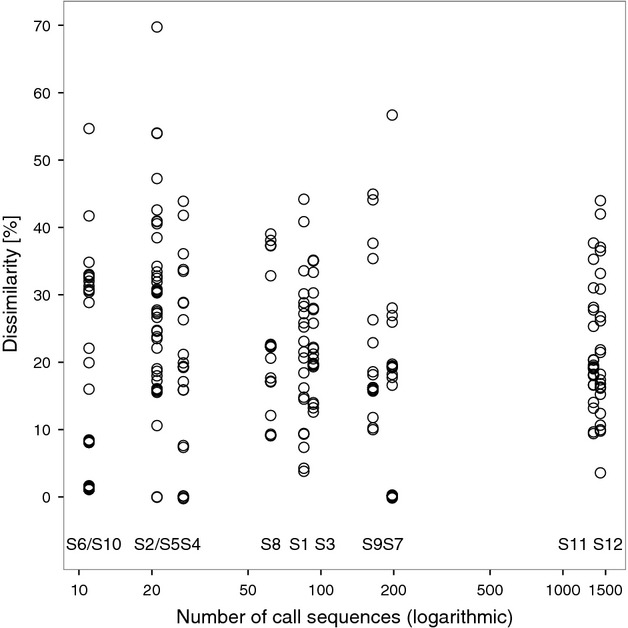
Percent dissimilarity (PERMDISP values) of the validated species lists for 12 sites produced by 21 operators (circles) in relation to the number of call sequences recorded in a study on operator bias in bat call identification. Each vertical strip represents a site (S1 to S12). Note the logarithmic scale of the abscissa. On two occasions, the same number of call sequences were recorded in two sites (S6/S10, S2/S5; 11 and 21 sequences).

The average heterogeneity of the species lists (median of the *z*_*ij*_ distances, Table 2) and the number of call sequences recorded at each site were related to each other in a nonlinear fashion: The *z*_*ij*_ declined with increasing sequences up to approximately 200 call sequences and then remained constant in the range of *z*_*ij*_ ∼20% dissimilarity (Fig. 6).

**Table 2:** PERMDISP analysis as a measure of the average variance of assemblages (% dissimilarity) for each site in a study on operator biases in bat call identification. Medians are calculated from the operators' individual PERMDISP values. The sites (S1–S12) are ranked by ascending number of call sequences (Fig 3). “batIdent” gives the unvalidated number of species from automated classification

Sites	S6	S10	S2	S5	S4	S8	S1	S3	S9	S7	S11	S12
Dissimilarity (median, %)	1.4	29.0	30.2	27.0	19.7	22.4	21.7	21.1	16.0	18.0	19.0	18.1
batIdent (number spec.)	2	7	6	5	5	9	10	7	10	7	14	12
Call sequences	11	11	21	21	27	62	85	93	164	197	1338	1429

## Discussion

Our results suggest that the operator bias introduced during the validation of results from software-aided bat call identification can be considerable and should be acknowledged especially in studies in which more than one person carries out validation. The operator-related differences in the final validated species lists were influenced by the amount of recorded material, operators' experience, and the difficulty of identifying the respective species.

Although we did not know the actual identity of the species in the empirical data set, our results indicate that the operators generally felt able to remove possible false positives and furthermore detected additional species that had been rejected in the automated process.

It is inherent to the batIdent algorithm that with increasing numbers of call sequences, the likelihood to produce false positives increases as well (see rates in the confusion table, Marckmann and Runkel 2010b). In the present study, unvalidated species lists showed a steady ascent in number of identified species and did not seem to reach an asymptote. At the same time, the curve of the validated species lists flattened out (Fig. 3). This was supported by comparing the homogeneity of assemblages as a whole. The dissimilarity of species lists decreased with increasing numbers of call sequences indicating that in general, similar species have been eliminated as possible artefacts from automated classification (Fig. 6, Table 2).

As a result of the software's systematic overestimation of species richness, the validated species lists produced by the operators were, in most cases, shorter than the unvalidated species list.

We concluded that validation of the raw data can successfully reduce excessive numbers of species, and thus, manual validation of the software output is indispensable.

All operators were volunteers who were aware that their work was part of a comparative study on biases in call analysis, so they were probably motivated. We cannot simply extrapolate from this to their everyday routine. However, it is reassuring that the operators in general questioned the output of the software and regarded the species lists produced by batIdent just as a baseline.

As we were not able to investigate individual false positives resulting from validation, other studies suggest that they do exist. Farmer et al. (2012) found at least one identification error in 73% of their testing scenarios, regardless of the observer's skill level, although overall proportions of false positives significantly increased as skill level decreased. The majority of these false positives were rare species. Miller et al. (2011) state that biases resulting from false positive errors are greatest when species are rare.

Royle and Link (2006) emphasized that even low false positive error rates can introduce extreme bias in estimates of site occupancy and can lead to substantial overestimation of occupancy probability.

We were surprised that many operators put additional species on their lists, most frequently *M. emarginatus*, *M. myotis*, *E. serotinus*, *P. nathusii*, *V. murinus,* and *Plecotus* spp. (Table 1). Operators discovered these species after in-depth examination of the recordings. Many of them are rare and had been identified by batIdent for only a few call sequences. Species were most frequently added to the lists by operators with intermediate and least experience. Adding species means scrutinizing the original data closely, and perhaps investigating individual calls using sound analysis software, so we conclude that operators who added species invested much effort in their analysis.

In apparent contrast to this, we could not find convincing evidence that investing more time generally increased species richness. This may be because additional effort can also be spent to eliminate doubtful species more confidently. Besides, manually checking for wrongly measured calls, that is, species wrongly classified, is not practicable for all species equally.

The OTUs Pmid and Mbart were frequently accepted by many operators with all levels of experience, although the species included are by no means straightforward to identify (cf. Hammer et al. 2009). For Pmid, the majority of operators did not trust the discriminative ability of the software and thus chose to accept only the upper-level epitheton. On the other hand, some other operators (of all experience levels) did identify *P. nathusii* and *P. kuhlii* as separate entities. Either these operators did believe that the batIdent's identifications were correct (the algorithm's false positive rate of *P. nathusii* is only 0, 21%) or they gave weight to biogeographical data: *P. nathusii* is found in a wider region than *P. kuhlii* (Dietz et al. 2009).

The high acceptance rate of Mbart (*M. mystacinus/brandtii*), the other “difficult” epitheton, was probably an effect of its high frequency in the unvalidated batIdent species lists. These species are part of an aggregate of small- and medium-sized species of the genus *Myotis* that produce extremely similar echolocation calls. In our experience, they are highly active in almost all forest or near-forest habitats and are rarely satisfactorily differentiated. Mbart is frequently the taxon recorded with highest dominance and therefore usually preferred by batIdent, while others (most notably, the rare *M. bechsteini*i) are dismissed in doubt.

All in all we could confirm the results of Miller et al. (2012), who suggest incorporating operator ability as a predictor of among-observer error rates. Validation patterns for operators with intermediate and most experience were distinctly different. The former produced longer species lists with large within-site variability, while the latter assessed conservatively producing short species lists.

In contrast to the considerable variability in the validation of species lists, caused predominantly by additional species, the dissimilarity of the species lists the operators produced for each site was around 20%. Most species lists could be well differentiated, irrespective of which operator had validated the data. Thus, if the characterization of bat assemblages is the goal of a study, operator bias is less of a concern than in species-specific investigations. However, this might not be true for sites where very few call sequences were recorded, as we found a negative correlation between the number of sequences and assemblage heterogeneity. At sites with little activity, batIdent can nevertheless produce rather long species lists, causing divergent validation behavior by operators. Vice versa, the more activity we recorded at a site, the less divergent were the operators' species lists, hence, the lower the operator bias.

The questionnaire results revealed that the operators rated the tools and resources we asked about very differently. There was no single item that operators unanimously ranked very high or very low (the most notable exception is the much appreciated graphical display of individual calls in bcAdmin, question 10g).

Of the supplements provided for site characterization (questions 9a to h), the aerial and site photographs and the large-scale (1:50,000) geographic map scored highest. This makes sense, as these resources inform the operator about the habitats at the site. Given the pronounced preferences of many Central European bat species, habitat information may provide valuable clues for accepting or declining species on the unvalidated lists.

Regarding software functions (questions 10a to j), the operators focused on the many “quality indicators” the software offers, for example, the displays of time–frequency diagrams of individual calls, the classification probability of calls within a sequence (higher probabilities are more trustworthy), and the number of calls per sequence (identification of long sequences with many calls is more reliable than identification of sequences with few, potentially distorted, calls). Many operators also consulted sound analysis software outside the batcorder system to verify classifications independently (11b) and call analysis literature (e.g., Russo and Jones 2002; Skiba 2003; Obrist et al. 2004; Hammer et al. 2009).

Thus, for validation, the “average operator” relied on the various software functions indicative of identification quality, used external software and literature, and checked species against supplements provided for site characterization. Bearing in mind the diversity among the operators, they took a very reasonable approach.

The identification bias demonstrated in this study is a major issue for broadscale and long-term studies in which more than one operator carries out validation. Thus, many inventories, surveys, and monitoring programs face this problem, and measures should be taken to standardize the validation process and calibrate the operators involved. This confirms the conclusions of Fitzpatrick et al. (2009) and Farmer et al. (2012), who showed that a heterogeneous mixture of operator skill levels can lead to biased results.

Elaborate quality control concepts used for other organisms (e.g., Sykes and Lane 1996; Wilkie et al. 2003; Sachteleben and Behrens 2010) may well be adopted for bat investigations: for instance, preparative joint workshops to agree on protocols/standard procedures/tools; a priori identification of possible error sources; preparation of identification guidelines and examples; procedures for dealing with difficult species/doubtful classifications; consistent documentation on decision criteria; regular meetings to discuss for example doubtful classifications; and peer review of doubtful identifications.

We found considerable differences in validation behavior between operators with different levels of expertise. From this and from our own experience, we suggest that only experienced operators (that is, people with experience of analyzing recordings made over a few hundred nights) validate bat calls in a consistent, invariant way. Jennings et al. (2008) found that operators with <1 years' experience performed worse than those with more experience. We suspect that validation behavior, especially of the least experienced operators, evolves in the course of an investigation. So, observer bias may occur even in investigations in which validation is carried out only by one person, as that person's level of experience increases over time. If the amount of recorded material is substantial, this may introduce bias. We therefore strongly recommend controlling for this in the experimental design, for instance by validating recordings in random or alternating order instead of sequentially or by validating the same subset of recordings several times in the course of the study and comparing the outcome.

Automated call analysis naturally lends itself to broadscale studies, but is also very useful for short-term investigations with limited resources, such as environmental impact assessments. However, the convenience and speed of automatic identification may result in underestimation of the importance of critical and informed validation, so that few resources are allocated to this part of the project. The relation between operator bias and experience in this study should act as a warning in this respect: Inexperienced biology students and other unskilled workers may be inexpensive, but they are very unlikely to deliver unbiased results. This study suggests that the validation of automatically analyzed bat calls, like the identification of other animals, is a job for experts.

## Supporting Information

**Appendix S1.** Questionnaire circulated to the volunteer operators.
