# Supplementary material for: Operator bias in software-aided bat call identification
Source: Ecol Evol. 2014 May 30;4(13):2703–13. doi: 10.1002/ece3.1122 (PMC4113294; doi:10.1002/ece3.1122)
Supplement: Supplementary file 1 — Appendix S1. Questionnaire circulated to the volunteer operators. [file ece30004-2703-SD1.doc]

**Supporting Information**

**Appendix S1.** Questionnaire circulated to the volunteer operators

Part 1: Details on the operator

| Name: | Date: |
| --- | --- |
| 1) How long have you already been involved in bat research? | |
|  | years |
| 2) Have you been engaged in detection and identification of bat calls, previous to this study? | |
| Yes / No | (delete non-applicable) |
| 3) How many nights (estimated) have you already worked with ultrasound detectors, batcorder, etc previous to this study? | |
|  | nights |
| 4) How many nights (estimated) have you already analysed using the batcorder system (ecoObs), previous to this study? | |
|  | nights |

Part 2: Details on the operators individual validation procedure

| 8) Information on the software | Version |
| --- | --- |
| bcAdmin |  |
| batIdent |  |
| R |  |
| In case settings have been changed in batIdent | Value |
| Defaults: Probability threshold = 0.6; Min. number of calls = 3  (see batIdent manual, p8, 3.3 Defaults – Preferences, http://www.batident.eu/Manual-batIdent.pdf) | |
| Probability threshold (Min. avg. probability for species extraction) |  |
| Min. number of calls (Min. call count for species extraction) |  |
| Outlier-thresholds | yes / no |
| In case of 'yes', for which species |  |
| In case settings have been changed in bcAdmin | Value |
| Defaults: Analysis threshold = -27dB; Quality = 20 (cf. bcAdmin2 manual, p13, p21, p35f; http://ecoobs.de/bcAdmin/Manual-bcAdmin2.pdf) | |
| Analysis threshold [dB] |  |
| Quality |  |
| 9) How useful was the supplementary site characterisation for validation? (1=not at all; 5=very) | |
|  | Rating (1 to 5) |
| site photographs |  |
| areal photograph |  |
| detail from a geographic map 1:50,000 |  |
| geographical coordinates and elevation |  |
| date of recordings |  |
| on-site ambient temperature records |  |
| additional climate data |  |
| site descriptions |  |
| 10) How important for your validation were single functions of batIdent / bcAdmin? (1=not at all; 5=very) | |
|  | Rating (1 to 5) |
| batIdent: |  |
| history |  |
| console |  |
| bcAdmin: |  |
| number of calls per call sequence |  |
| duration of the call sequence |  |
| classification probability (%) of call sequence |  |
| classification probability (%) of single calls |  |
| graphical representation of single calls |  |
| diagram nightly activity |  |
| diagram identification tree |  |
| diagram identification quality |  |
| others: |  |
| 11) Which additional documentation and tools did you use during validation and which significance did they have for the affirmation of the results? (1=not important; 5=very important) | |
|  | Rating (1 to 5) |
| collection of bat call samples |  |
| call analysis software (e.g.: Batsound, bcAnalyze, …) |  |
| personal records |  |
| distribution maps |  |
| general literature on biology, habitat, ... (e.g. Dietz) |  |
| specialised literature on calls & call analysis (e.g. Skiba, Hammer & Zahn, …) |  |
| specialised literature on batcorder (e.g. Marckmann) |  |
| others: |  |
